# Correlation analysis of sagittal alignment and skeletal muscle mass in patients with spinal degenerative disease

**DOI:** 10.1038/s41598-018-33867-0

**Published:** 2018-10-19

**Authors:** Akihiko Hiyama, Hiroyuki Katoh, Daisuke Sakai, Masato Sato, Masahiro Tanaka, Tadashi Nukaga, Masahiko Watanabe

**Affiliations:** Department of Orthopaedic Surgery, Tokai University School of Medicine, 143 Shimokasuya, Isehara, Kanagawa 259–1193 Japan

## Abstract

We investigated how skeletal muscle mass (SMM) affects spinal sagittal balance (radiographic parameters) in symptomatic spinal patients. The first purpose of this study was to evaluate the body composition and the spinal sagittal alignment in symptomatic spinal patients. The second purpose of this study was to compare whether the body composition and the spinal sagittal alignment is different in patients with cervical spine disease and lumbar spine disease. We retrospectively evaluated 313 patients who were hospitalized for surgery to treat spinal degenerative disease, who were divided into cervical and lumbar spine disease groups. All patients underwent full-length standing whole-spine radiography and bioimpedance analysis (BIA) before surgery. We used standard measurements to assess the sagittal vertical axis (SVA), cervical lordosis (CL; C2–C7), lumbar lordosis (LL; T12–S1), thoracic kyphosis (TK; T5–12), pelvic incidence (PI), pelvic tilt (PT), and sacral slope (SS). We also analyzed radiological and body composition parameters, patient characteristics, and the correlation between SMM and each sagittal parameters. In the overall cohort, the mean age at the time of operation was 66.5 ± 15.3 years and 59.2% of the patients were men. The correlation coefficients (r) between SMM and PT were negative weak correlation (r = −0.343, P < 0.001). The correlation with SMM for other LL, PI, SS, and SVA was statistically significant, but the correlation was none. In addition, our results also suggested strong correlations (r > 0.5) between LL and SS (r = 0.744), between LL and SVA (r = −0.589), between PT and SS (r = −0.580), and LL and PT (r = −0.506). Fifty-seven patients (18.2%, cervical group) had cervical spine disease and 256 patients (81.8%, lumbar group) had lumbar spine disease. No significant differences in age, height, body weight, and body mass index were observed between the two groups. The SMM of patients with cervical and lumbar spine disease also did not differ significantly. In the lumbar group, correlations were found between SMM and PT (r = −0.288, *P* < 0.001), between SMM and LL (r = 0.179, *P* < 0.01), and between SMM and SS (r = 0.170, *P* < 0.01), while only PT (r = −0.480, *P* < 0.001) was negatively correlated with SMM in the cervical group. This analysis indicated that PT is the sagittal parameter most closely related to SMM in patients with the spinal degenerative disease. The SMM might be one of the important factors that influenced the posterior inclination of the pelvis in symptomatic spinal patients, especially in cervical spine disease.

## Introduction

Sarcopenia is defined as the age-associated loss of skeletal muscle mass (SMM) and function with a risk of adverse outcomes such as physical disability and poor quality of life (QOL)^1^. Sarcopenia is very common in older individuals, with a reported prevalence of 60 to 70-year-olds of 5–13%^2^. An analysis of the prevalence of sarcopenia by disease reported that it occurred in 4/25 cases (16%) of lumbar spinal canal stenosis and 7/15 cases (46.6%) of degenerative lumbar spine disease, indicating a high prevalence in degenerative lumbar spine disease^3^.

Multiple studies have described normative values for parameters of spinopelvic alignment in different populations of varying ages, and pathologic conditions.

The sagittal alignment of the spine is also thought to be one of the most important factors influencing disorders of the neck and the lower back. Especially in older individuals, in whom the spinal sagittal alignment is likely to be anteversion, spinal sagittal alignment is strongly associated with health-related quality of life (QOL)^4^. It has recently been reported that QOL deteriorates not only because of lumbar spine and pelvic malalignment but also because of cervical deformity^5,6^. Generally, sagittal imbalance results in increased muscular effort and energy expenditure, causing pain, fatigue, and disability. Muscle mass decreases with age. Observational studies have shown an annual decline of approximately 1% after the age of 40^7^. Due to muscular weakness, older individuals have difficulties in supporting their weight on the lower extremity, thereby causing disabilities in balance. Therefore, the relationship between the mass of trunk muscle, including skeletal muscle, and low back pain has also attracted attention. Some researchers have reported an association of body composition with musculoskeletal pain and have demonstrated that a greater fat mass and an attenuated muscle mass were associated with increased musculoskeletal pain^8,9^.

We think that sagittal balance understanding is a primordial factor in implementing an accurate surgical strategy in spinal degenerative disease. The previous studies have demonstrated that cervical sagittal alignment is correlated with thoracolumbar and pelvic alignments in asymptomatic patients and young idiopathic scoliosis patients^10,11^. However, there are few relational studies on body composition and spinal alignment of symptomatic spinal diseases. In addition, there also has been no report comparing whether the body composition or the spinal sagittal alignment differs for the patient with cervical spine disease and lumbar spine disease.

Therefore, it is important to analyze their SMM and spine sagittal alignment, as people who are actually treated are symptomatic patients.

Considering these issues, we investigated how SMM affects spinal sagittal balance (radiographic parameters) in symptomatic spinal patients. SMM means appendicular muscle including arm and leg. These muscles are considered not directly involved in spine alignment. However, improvement by nutritional guidance and muscle exercise is not trunk muscles but whole body SMM. Therefore, we think that analyzing how whole body SMM affects spine alignment will give new findings. The first purpose of this study was to evaluate the body composition and the spinal sagittal alignment in symptomatic spinal patients. The second purpose of this study was to compare whether the body composition and the spinal sagittal alignment is different in patients with cervical spine disease and lumbar spine disease.

In addition, we hope that this study will enhance the understanding of the sagittal plane of the spine in symptomatic spinal patients and the importance of muscle strengthening exercise programs in patients with the spinal degenerative disease.

## Results

The demographics of the 313 patients are listed in Table 1. In the overall cohort, the mean age at the time of operation was 66.5 ± 15.3 years and 58.8% of the patients were men. The average body mass index (BMI) was 24.0 ± 4.0 kg/m^2^. The mean value and SD of spino-pelvic parameters are listed in Table 2. Regarding the normal value of Japanese populations according to Asai *et al*.^12^, sagittal vertical axis (SVA) and pelvic tilt (PT) was larger and lumbar lordosis (LL; T12–S1) and thoracic kyphosis (TK; T5–12) were smaller. Pelvic incidence (PI) was almost the same.

**Table 1:** Summary of characteristics in 313 study patients.

	Overrall cohort
Number of Cases	313
Gender (Men: Women)	M184 W129
Mean Age ± SD (yrs)	66.5 ± 15.3
Height (cm)	160.4 ± 9.4
Body weight (kg)	62.3 ± 14.4
BMI (kg/m2)	24.0 ± 4.0
ICW (ℓ)	19.9 ± 4.6
ECW (ℓ)	12.8 ± 2.7
Protein (kg)	8.6 ± 2.0
Mineral (kg)	3.1 ± 0.6
Soft lean mass (kg)	41.7 ± 9.4
Skeletal muscle mass (kg)	24.0 ± 6.0
Body fat mass (kg)	17.8 ± 8.4
Percent body fat (%)	27.7 ± 9.5
Waist Hip Ratio	0.881 ± 0.069

BMI, body mass index,ICW, intracellular water; ECW, extracellular water.

**Table 2:** Detailed sagittal parameters of the subjects and correlation analyses.

Radiological parameters	Overrall cohort	Asai *et al*.^12^
CL	9.1 ± 13.8	—
TK	24.8 ± 11.5	M38.5 ± 10.7 F37.2 ± 12.8
LL	31.9 ± 18.8	M44.5 ± 12.7 F45.9 ± 14.0
PI	49.8 ± 9.8	M47.7 ± 9.9 F51.2 ± 10.8
PT	23.5 ± 10.0	M15.8 ± 7.6 F19.5 ± 9.7
SS	25.9 ± 10.4	—
SVA	71.2 ± 74.3	M12.7 ± 41.3 F10.1 ± 43.4

Summary of sagittal parameters in 313 study patients.

SMM; skeletal muscle mass; CL, cervical lordosis (C2–C7); TK, thoracic kyphosis (Th5–12); LL, lumbar lordosis (T12–S1); PI, pelvic incidence; PT, pelvic tilt; SS, sacral slope; SVA; sagittal vertical axis.

Table 3 shows that lumbar canal stenosis was the most prevalent diagnosis in the lumbar group, while cervical myelopathy was the most prevalent in the cervical group.

**Table 3:** Diagnosis of spinal degenerative disease in 313 study patients.

Diagnosis	No. of patients
Cervical myelopathy	32
Cervical OPLL	11
Cervical disc herniation	6
Atlanto-axial subluxation	4
Dropped head syndrome	2
Cervical spondylolisthesis	2
**Cervical group**	**57**
Lumbar canal stenosis	128
Lumbar disc herniation	48
Adult spinal deformity	39
Spondylolisthesis/Spondylolysis	32
Dialysis associated spondylosis	4
Other disease	5
**Lumbar group**	**256**

Fifty-seven patients (18.2%, cervical group) had cervical spine disease and 256 patients (81.8%, lumbar group) had lumbar spine disease. No significant differences in age, height, body weight and BMI were observed between the two groups (Table 4). There were no significant differences in biochemical and bioimpedance parameters between the two groups (Table 4).

**Table 4:** Detailed body compositions analysis, muscle-fat analysis, obesity estimation, and body water analysis in the two groups.

	Cervical group	Lumbar group	*p value*
Number of Cases	57	256	
Gender (Men: Women)	M42 W15	M142 W114	0.012*
Mean Age ± SD (yrs)	66.2 ± 12.2	66.5 ± 16.0	*0.349*
Height (cm)	162.0 ± 9.7	160.0 ± 9.4	*0.123*
Body weight (kg)	63.5 ± 15.7	62.0 ± 14.1	*0.321*
BMI (kg/m^2^)	24.1 ± 4.4	24.0 ± 3.9	*0.708*
ICW (ℓ)	20.7 ± 4.8	19.8 ± 4.5	*0.097*
ECW (ℓ)	13.2 ± 2.8	12.8 ± 2.7	*0.257*
Protein (kg)	9.0 ± 2.1	8.5 ± 1.9	*0.098*
Mineral (kg)	3.2 ± 0.7	3.1 ± 0.6	*0.061*
Soft lean mass (kg)	43.4 ± 9.8	41.4 ± 9.3	*0.095*
Skeletal muscle mass (kg)	25.0 ± 6.3	23.8 ± 5.9	*0.098*
Body fat mass (kg)	17.9 ± 8.9	17.7 ± 8.4	*0.768*
Percent body fat (%)	26.9 ± 10.0	27.9 ± 9.4	*0.642*
Waist Hip Ratio	0.877 ± 0.070	0.882 ± 0.069	*0.972*

ICW, intracellular water; ECW, extracellular water.

There were significant differences in cervical lordosis (CL; C2–C7) (*P* < 0.05), LL (*P* < 0.001), PT (*P* < 0.001), SS (*P* < 0.01), and SVA (*P* < 0.001) between the groups. However, TK (*P* = 0.189) and PI (*P* = 0.089) did not differ significantly between the two groups (Table 5).

**Table 5:** Detailed sagittal parameters of the subjects in the two groups (Cervical group and lumbar group).

Radiological parameters	Cervical group	Lumbar group	*p value*
CL	4.7 ± 14.5	10.1 ± 13.5	0.014*
TK	26.0 ± 10.5	24.5 ± 11.7	*0.189*
LL	40.0 ± 20.3	30.2 ± 18.0	0.000***
PI	48.0 ± 9.3	50.2 ± 9.8	*0.089*
PT	18.3 ± 10.4	24.6 ± 9.6	0.000***
SS	29.5 ± 12.7	25.2 ± 9.7	0.001**
SVA	45.5 ± 75.6	76.6 ± 73.0	0.000***

CL, cervical lordosis (C2–C7); TK, thoracic kyphosis (Th5–12); LL, lumbar lordosis (T12–S1); PI, pelvic incidence; PT, pelvic tilt; SS, sacral slope; SVA; sagittal vertical.

^**^<0.01, ^***^<0.001 indicates significant differences between groups.

**Figure 1:**
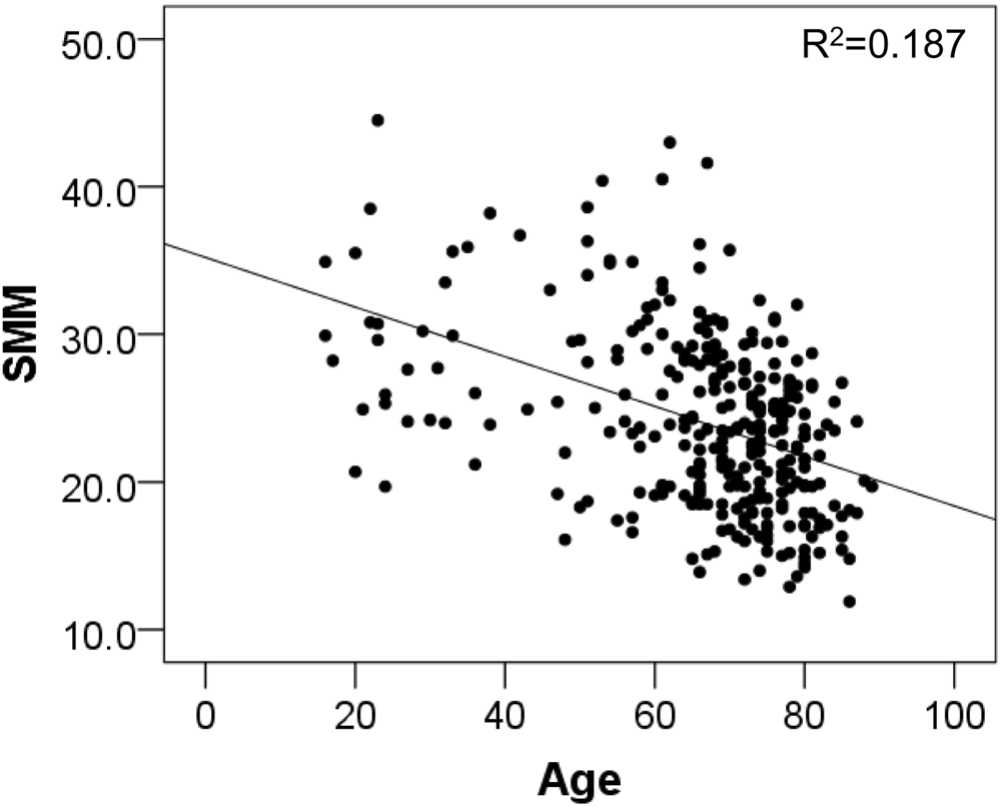
Correlation between SMM and age in patients with spinal degenerative disease (n = 313) SMM; Skeletal muscle mass.

Analysis of the correlation between age and SMM of the 313 patients showed a negative correlation (r = −0.423, P < 0.001) (Fig. 1).

**Figure 2:**
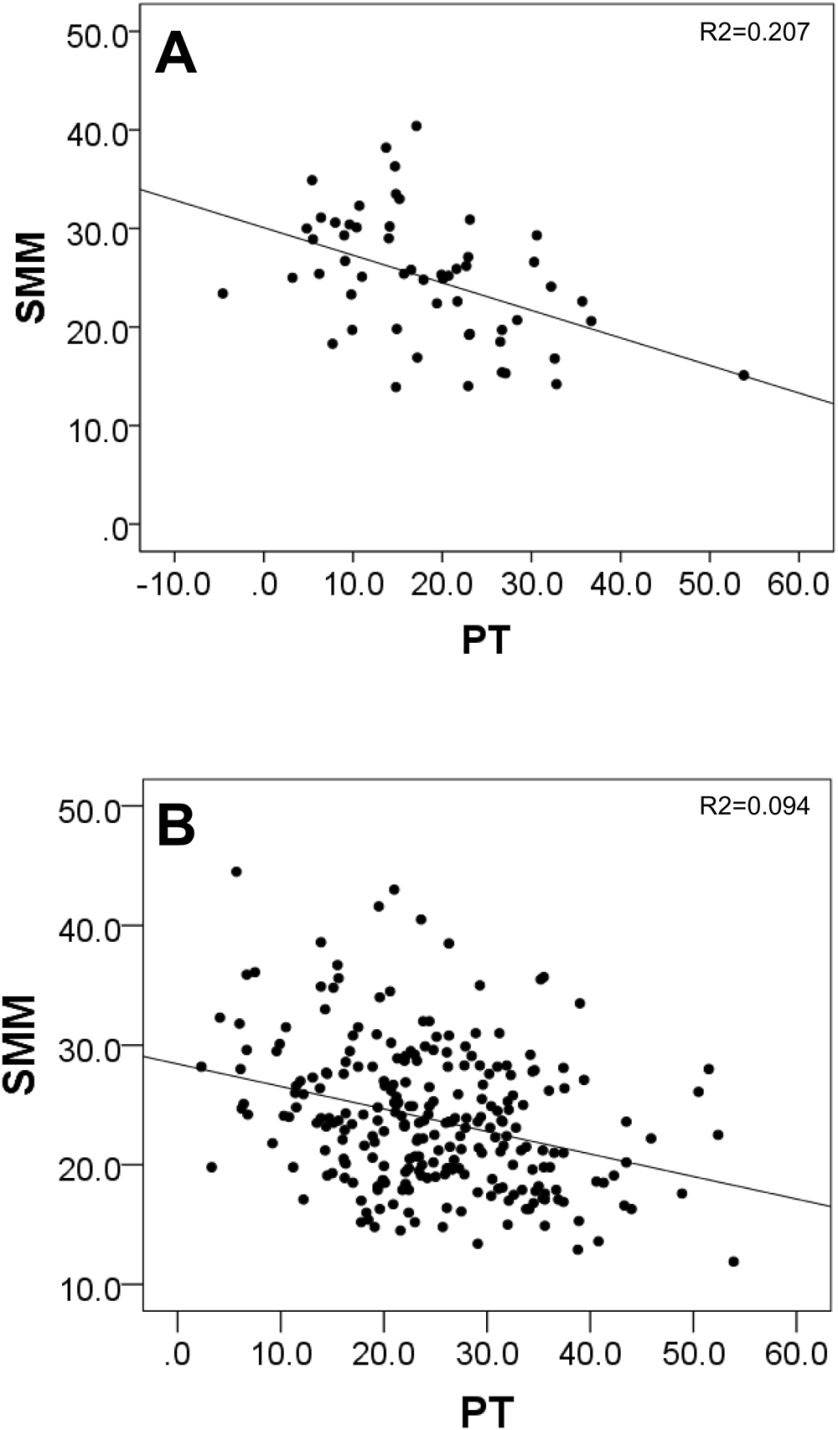
(**A**) Correlation between SMM and PT in patients with degenerative cervical spine disease (n = 53). (**B**) Correlation between SMM and PT in patients with degenerative lumbar spine disease (n = 251) A significant negative correlation between SMM and PT was noted. SMM; Skeletal muscle mass, PT; pelvic tilt.

Analysis of the correlation between SMM and each sagittal parameter was also performed. The correlations with SMM were found for PT (r = −0.288, *P* < 0.001), LL (r = 0.179, *P* < 0.01), SS (r = 0.170, *P* < 0.01), PI (r = −0.167, *P* < 0.01) and SVA (r = −0.163, *P* < 0.01) in the lumbar group, while only PT (r = −0.480, *P* < 0.001) was negatively correlated with SMM in the cervical group. These results demonstrated that PT was most negatively correlated with SMM in patients with cervical or lumbar spine disease. Further, SMM and PT were more correlated in patients with cervical spine disease than patients with lumbar spine disease (Table 6). Figure 2 shows a scatter plot of SMM and PT in patients with cervical (Fig. 2A) or lumbar spine disease (Fig. 2B). The results demonstrate a significant negative correlation between spinal alignment (PT) and SMM.

**Table 6:** Spearman correlations mean (Spearman’s *r*) between SMM and radiological parameters.

	SMM	CL	TK	LL	PI	PT	SS	SVA
**Cervical group (n** = **57)**								
SMM	1.000	−0.006	−0.035	0.202	−0.220	−0.480***	0.194	−0.248
CL	−0.006	1.000	0.243	0.198	0.257	0.005	0.292*	0.293*
TK	−0.035	0.243	1.000	0.402**	0.077	−0.094	0.219	0.007
LL	0.202	0.198	0.402**	1.000	0.307*	−0.515***	0.795***	−0.248
PI	−0.220	0.257	0.077	0.307*	1.000	0.306*	0.486***	0.247
PT	−0.480***	0.005	−0.094	−0.515***	0.306*	1.000	−0.584***	0.121
SS	0.194	0.292*	0.219	0.795***	0.486**	−0.584***	1.000	0.105
SVA	−0.248	0.293*	0.007	−0.248	0.247	0.121	0.105	1.000
**Lumbar group (n** = **256)**								
SMM	1.000	−0.088	−0.044	0.179**	−0.167**	−0.288***	0.170**	−0.163**
CL	−0.088	1.000	0.227***	−0.110	0.079	0.172**	−0.088	0.288***
TK	−0.044	0.227***	1.000	0.416***	0.106	−0.045	0.125*	0.030
LL	0.179**	−0.110	0.416***	1.000	0.257***	−0.457***	0.718***	−0.624**
PI	−0.167**	0.079	0.106	0.257***	1.000	0.454***	0.409***	0.086
PT	−0.288***	0.172**	−0.045	−0.457***	0.454***	1.000	−0.538***	0.347***
SS	0.170**	−0.088	0.125*	0.718***	0.409***	−0.538***	1.000	−0.306***
SVA	−0.163**	0.288***	0.030	−0.624**	0.086	0.347***	−0.306***	1.000

Cervical group (n = 57).

Lumbar group (n = 256).

SMM; skeletal muscle mass; CL, cervical lordosis (C2–C7); TK, thoracic kyphosis (Th5–12); LL, lumbar lordosis (T12–S1); PI, pelvic incidence; PT, pelvic tilt; SS, sacral slope; SVA; sagittal vertical axis ^*^p < 0.05, ^**^<0.01, ^***^<0.001 indicates significant differences between groups.

## Discussion

In this study, we found that decreases in SMM were most strongly associated with posterior PT, and an increased PT and smaller SMM may be more common in symptomatic patients. This indicates that of the sagittal parameters, PT has the most important relationship with SMM in patients with cervical spine disease. There is increasing recognition of the importance of spinal sagittal alignment in relation to LBP. Today, it is clear that aging and degeneration are kyphosis process in which LL diminishes and TK increases. With increasing age, SVA, TK, and PT tend to increase, whereas LL tends to decrease^13^. It has been suggested that a decrease in SMM may have the greatest effect on spinal alignment leading to a posterior PT.

Sagittal alignment is well known to be correlated with QOL in patients with DLS disease. Schwab *et al*. proposed a systematic evaluation method to classify sagittal spinal alignment in flat-back cases with regard to its adverse effect on QOL^14^. Their study demonstrated that SVA, PT, and PI–LL were most closely related with LBP and disability. Glassmann *et al*. found that patients with a large SVA suffered the greatest disruption in QOL, which emphasized the importance of sagittal alignment^4^. Lafage *et al*. also associated posterior PT and stooping posture with poor QOL and therefore considered PT and SVA to be vital factors.

Furthermore, preoperative PI–LL and symptom duration were independently associated with SVA improvement in lumbar spine canal stenosis patients with a forward-bending posture^15^.

There are numerous studies aimed at improving our understanding of the ideal global sagittal alignment, including that of the pelvis. Since the work by Duval-Beaupére *et al*., several studies have attempted to clarify whether PI is of primary importance in regulating sagittal alignment, although the relationship between LL and PI is accepted^16,17^. PT, a dynamic pelvic parameter reflecting pelvic retroversion, must be considered because increased PT implies residual postoperative spinal deformity and negatively affects function and thus postoperative outcomes. However, the goal of surgery is to achieve optimal sagittal alignment by restoring an optimal LL, not PT, for patients with DLS diseases.

Hasegawa *et al*. examined the normative values for all parameters of the standing sagittal alignment and balance including the head and lower extremities in a cohort of 126 healthy Japanese adult volunteers using a new scanning X-ray imaging system (EOS imaging, Paris, France). They reported that a linear regression analysis of standard spinal parameters and PI–LL showed that PT was most significantly correlated with PI–LL. Furthermore, the Oswestry Disability Index score also showed a trend towards a positive correlation^18^. These results demonstrate the importance for pain and QOL of posterior PT in spinal alignment. Compared to the thoracic and lumbar spine, the guidelines for the assessment of the sagittal alignment of the cervical spine have not been clearly defined.

Limitations of this study include the small number of participants and the lack of a control group. This study only compared patients with spinal degenerative disease and did not compare these patients to a normal population without back problems. Because this is a symptomatic patient who actually has clinical problems, we evaluated these patients in this study. However, future studies with more participants and a control group are needed to confirm the findings of our study. Second, the study was a cross-sectional analysis, not a longitudinal one, so we did not analyze postoperative spinal alignment. Because PI–LL mismatch and large PT have been implicated in proximal junctional kyphosis after corrective surgery, this should be investigated further^19,20^. Furthermore, we only prove the relevance between PT and SMM, Unfortunately, we do not know why patients with cervical spine disease have a high correlation with PT as compared with patients with lumbar spine disease, we think that it is important to establish a causal relationship in the future. The third limitation of the study was the use of the bioimpedance analysis (BIA) method to measure body composition rather than dual-energy X-ray absorptiometry (DXA), which is regarded as the most reliable tool to evaluate body composition and is considered to be the gold standard in clinical practice. BIA has a tendency to overestimate muscle mass compared with DXA, but the agreement between DXA and BIA is high for lean arm mass and for axial lean mass^21^. In addition, a study by Spungen *et al*. reported a meaningful correlation between the values measured by BIA and DXA. Therefore, our assumption of the appropriateness of body composition measured by BIA in this study is well suited for evaluating in our patients^22^. The forth limitation of this study was that considering the influence of gender on the fat content of the body, which would impact the body water measurements, but this time analysis did not do the comparison by gender. The final limitations of this study are that it was a single-center retrospective study conducted at a tertiary care university hospital, which limits its generalizability, and that the number of subjects was small. The spectrum of presenting patients obviously differs between primary care clinics, community hospitals, and tertiary care facilities. A multicenter study including a spectrum of medical facilities spanning both metropolitan and rural areas may resolve this limitation.

## Conclusions

We investigated how SMM affected spine alignment for patients with the spinal degenerative disease. SMM in patients with spinal degenerative disease decreased with age, but there were no significant differences between patients with cervical and lumbar spine disease. Analysis of the correlation between spinal alignment and skeletal muscle volume revealed that SMM and PT were the most strongly correlated. Therefore, we suggest that the decrease in SMM with age may be related to a posterior inclination of the pelvis. We believe that the increase in SMM and improvement in PT by nutritional guidance and muscle strengthening exercise program may reduce symptomatic spinal patients. However, we could not evaluate the relationship with QOL in this study. We believe that a prospective analysis is needed as to whether QOL will improve by increasing SMM or decreasing PT.

## Patients and Methods

### Ethics

The Committee on Ethics and the Institutional Review Board of Tokai University School of Medicine approved the study protocol (17R-237). All data from the patients were obtained in accordance with the revised Declaration of Helsinki (2013). We have obtained informed consent form with opt-out method from patients.

### Included patients

We retrospectively evaluated patients who were hospitalized for surgery to treat spinal degenerative disease from October 2015 to January 2018. The study group included 313 patients (184 men, 129 women) who were diagnosed with the spinal degenerative disease and had undergone BIA^21^ and whole-spine posteroanterior and lateral full-spine radiographs. The term “spinal degenerative disease” refers to any disease of the spinal column that results from the aging process and wear and tear that occurs to the bone, soft tissues and intervertebral disc of the spine in this study. Five spinal surgeons diagnosed spinal degenerative disease based on subjective symptoms, neurological findings, and magnetic resonance imaging.

The 313 patients were divided into those with cervical spine disease (cervical group) and those with lumbar spine disease (lumbar group).

X-ray evaluation involved examination of standing-erect whole-spine posteroanterior and lateral full-spine radiographs. For the lateral films, the patients stood with their knees locked and fully extended when possible, the feet shoulder-width apart, looking straight ahead, and with their elbows bent and knuckles in the supraclavicular fossa bilaterally. Body composition was measured using Inbody S20 (Biospace Inc., Seoul, Korea), which is a bedside body composition analyzer for patients who cannot stand.

The exclusion criteria were patients with: (1) metastatic tumor, (2) peripheral neuropathy, (3) spinal damage from trauma, (4) fresh vertebral compression fracture or (5) severe chronic depression that required the use of several antidepressant medications.

Clinical outcomes, radiological parameters, body composition analyses, and patient characteristics including sex, age, height, body weight, and BMI were examined. Analysis of the correlation between SMM and each sagittal parameter was performed for all patients.

### Radiological parameters

The radiographs were taken in the standing position. For the lateral films, the patients stood with their knees locked, the feet shoulder-width apart, looking straight ahead, and with their elbows bent and knuckles in the supraclavicular fossa bilaterally. The hip joint and cervical spine were included. All morphologic data were archived using picture archiving and communication systems (PACS). We used standard measurements reported elsewhere^23^ to assess sagittal balance (SVA), CL, LL, TK, PI, PT, and SS. The following variables were measured as follows—PI: defined as the angle between the perpendicular to the upper sacral endplate at its midpoint and the line connecting this point to the femoral head. PT: Angle between the vertical line and line joining hip axis to the center of superior endplate of S1. SS: Angle between superior endplate of S1 and horizontal line. CL: Cobb from the second cervical vertebral to 7th cervical vertebral. LL: Segmental angle of superior endplate of L1 and superior endplate of S1. TK: Cobb from the fifth thoracic vertebral to 12th thoracic vertebral (T5–T12). SVA: The horizontal distance between the posterior corner of the sacrum and the C7 plumb line. A positive value was defined when the sacral posterior corner landed in front of the C7 plumb line^14^. The data and measurements from the picture were assessed by 2 independent observers. After an agreement was reached between the observers, each parameter was independently measured twice by 2 spinal surgeons.

### Bioelectric impedance analysis

BIA has been used in various contexts for the measurement of the nutritional components of body composition, such as fat mass or fat-free mass, using the electrical properties of body tissues^24^. It has recently shown promise as a tool for the measurement of volume status^25,26^. The Inbody S20 analyzer measures the electrical responses at multiple frequencies between 1 and 1000 kHz and calculates the total body water (TBW), extracellular water (ECW), and intracellular water (ICW) by Chamney *et al*.’s method^25^. Inbody S20 was used according to the manufacturer’s guidelines. Briefly, the measurements were performed with the patient in the supine position using eight hand and foot tactile electrodes. We used the Touch Type electrode. The hand electrodes were positioned thumb and the middle finger. The foot electrodes were also positioned between examinee’s anklebone and heel.

The input variables included the patients’ age, sex, height, and actual body weight.

### Statistical analysis

Statistical analysis was performed using IBM SPSS Statistics version 20.0 (IBM Corp., Armonk, NY). All values are expressed as mean ± standard deviation (SD). An analysis of variance with a posthoc test (Mann–Whitney U test) was used for comparisons. The correlations between skeletal muscle mass and sagittal alignment were analyzed using Spearman’s product-moment correlation coefficient.

To identify the minimum number of participants required for adequate statistical power, we used the G-Power Analysis software program (G Power 3.1.9, University of Düsseldorf, Germany, http://www.gpower.hhu.de/)^27^.

The Spearman rank correlation coefficient (*r*) was used to examine correlations between variables of SMM and spino-pelvic parameters. The Spearman correlation coefficient was interpreted as follows: <0.3: none; 0.3–0.5: weak; 0.5–0.7: strong; 0.7–0.9: very strong; and >0.9: excellent.

A power analysis performed to calculate the minimum sample size necessary to detect a difference between two independent groups (calculated with Cohen’s d = 0.55, alpha = 0.05, two-tailed, power = 0.80) indicated a required sample size of 53 participants. A power analysis performed to calculate the minimum sample size necessary to detect a correlation (calculated with effect size = 0.3, alpha = 0.05, power = 0.90) indicated a required total sample size of 109 participants. For all statistical analyses, the type 1 error was set at 5% and p < 0.05 was considered significant.
